# Introduction of monkeypox vaccines; ahead of a looming pandemic

**DOI:** 10.1016/j.amsu.2022.104370

**Published:** 2022-08-18

**Authors:** Daniya Naveed, Hadia Nadeem, Muttia Abdul Sattar

**Affiliations:** Karachi Medical and Dental College, Pakistan

Monkeypox (MP), caused by monkeypox virus, is a rare endemic zoonotic disease found in tropical rainforest areas of the west or central Africa. MP is regarded as the most important Orthopoxvirus infection in human beings since the eradication of Smallpox [1], and it commonly presents with symptoms like high fever, body and muscle ache, enlarged lymph nodes and rash [2]. The transmission of MP from one person to another is through physical contact with blisters, scabs, body fluids, respiratory droplets, and contaminated surfaces such as clothing and bedding of the infected person. It can also be spread through the bite of an infected animal; particularly rats, mice and squirrels or by eating their raw or undercooked meat [2].

Since the emergence of MP in the 1970s, outbreaks have been reported in many countries, but mostly restricted to endemic areas. In early May 2022, MP cases were reported for the first time in UK, Spain and elsewhere in Europe [3], Thereafter, as per a CDC report on 5th August 2022, there have been 28 220 confirmed cases of MP worldwide, out of which 27 875 cases were reported in 81 different countries that have not historically reported MP [4]. The spike in MP cases in non-endemic regions is worrisome and on 4th August 2022, the US government declared MP as a public health emergency [5].

Two vaccines are currently approved in the U.S that can protect against MP, the JYENNEOS vaccine and the ACAM2000, licensed by U.S Food and Drug Administration (FDA). Prior smallpox vaccination may also result in milder illness [6]. The vaccines contain the live vaccinia virus. The attenuated form in JYENNEOS is administered as two subcutaneous injections 28 days apart and a maximum immune response is achieved within 14 days after the second dose. ACAM2000 is one percutaneous injection and takes 4 weeks to yield the outcome. It contains the replicating competent form of virus which poses a serious threat to people who are immunocompromised, have an exfoliative skin condition, are pregnant [6]. Vaccines serve as an advantage to be ahead of another looming pandemic in contrast to COVID-19. By inducing immunity ahead of infection and ensuring milder effects upon contact, it reduces the severity of consequences on the community. The COVID-19 pandemic already depicts desperation for vaccines, the economic downfall for which is yet to be overcome, one cannot afford another global crisis. Regardless of low mortality rates, there is no guarantee that the graph won't rise with new waves.

We advise the concerned authorities to start vaccination programs worldwide. The demand exceeding the supply is anticipated therefore a strategic plan must be formulated to cater for the endangered first which includes people living or travelling to heavily forested and rural areas of Central Africa, handling and preparing bush meat and healthcare workers. Homosexual men should receive health information and guidance regarding safe sexual practice without being stigmatized in any way. The general precautionary measures against COVID-19 should also protect against MP transmission. Risk communication should be informed by social listing insights. Moreover, new vaccines plans should be developed. To contain the virus, we need to act timely before it’s too late.

## Please state any sources of funding for your research

None.

## Ethical approval

None required.

## Consent

None required.

## Author contribution

Daniya Naveed, Hadia Nadeem, Muttia Abdul Sattar, All authors contributed the same.

## Registration of research studies

• Name of the registry:
• Unique Identifying number or registration ID:
• Hyperlink to your specific registration (must be publicly accessible and will be checked):

## Guarantor

None.

## Declaration of competing interest

None.
